# Age-dependence of healthcare interventions for COVID-19 in Ontario, Canada

**DOI:** 10.1186/s12889-021-10611-4

**Published:** 2021-04-12

**Authors:** Irena Papst, Michael Li, David Champredon, Benjamin M. Bolker, Jonathan Dushoff, David J. D. Earn

**Affiliations:** 1 Center for Applied Mathematics, Cornell University, Ithaca, USA; 2 Department of Biology, McMaster University, Hamilton, Canada; 3 South African Centre for Epidemiological Modelling and Analysis, University of Stellenbosch, Stellenbosch, South Africa; 4 Department of Pathology and Laboratory Medicine, Western University, London, Canada; 5 Michael G. DeGroote Institute for Infectious Disease Research, McMaster University, Hamilton, Canada; 6 Department of Mathematics & Statistics, McMaster University, Hamilton, Canada; 7 Department of Mathematics, University of Toronto, Toronto, Canada

**Keywords:** Epidemiology, Infectious disease, SARS-CoV-2, COVID-19, Age distribution, Hospitalization

## Abstract

**Background:**

Patient age is one of the most salient clinical indicators of risk from COVID-19. Age-specific distributions of known SARS-CoV-2 infections and COVID-19-related deaths are available for many regions. Less attention has been given to the age distributions of serious medical interventions administered to COVID-19 patients, which could reveal sources of potential pressure on the healthcare system should SARS-CoV-2 prevalence increase, and could inform mass vaccination strategies. The aim of this study is to quantify the relationship between COVID-19 patient age and serious outcomes of the disease, beyond fatalities alone.

**Methods:**

We analysed 277,555 known SARS-CoV-2 infection records for Ontario, Canada, from 23 January 2020 to 16 February 2021 and estimated the age distributions of hospitalizations, Intensive Care Unit admissions, intubations, and ventilations. We quantified the probability of hospitalization given known SARS-CoV-2 infection, and of survival given COVID-19-related hospitalization.

**Results:**

The distribution of hospitalizations peaks with a wide plateau covering ages 60–90, whereas deaths are concentrated in ages 80+. The estimated probability of hospitalization given known infection reaches a maximum of 27.8% at age 80 (95% CI 26.0%–29.7%). The probability of survival given hospitalization is nearly 100% for adults younger than 40, but declines substantially after this age; for example, a hospitalized 54-year-old patient has a 91.7% chance of surviving COVID-19 (95% CI 88.3%–94.4%).

**Conclusions:**

Our study demonstrates a significant need for hospitalization in middle-aged individuals and young seniors. This need is not captured by the distribution of deaths, which is heavily concentrated in very old ages. The probability of survival given hospitalization for COVID-19 is lower than is generally perceived for patients over 40. If acute care capacity is exceeded due to an increase in COVID-19 prevalence, the distribution of deaths could expand toward younger ages. These results suggest that vaccine programs should aim to prevent infection not only in old seniors, but also in young seniors and middle-aged individuals, to protect them from serious illness and to limit stress on the healthcare system.

## Background

In early 2020, the first outbreak of severe acute respiratory syndrome coronavirus 2 (SARS-CoV-2) was reported in Wuhan, Hubei Province, China [1]. The virus, which can cause the development of Coronavirus Disease 2019 (COVID-19), has been detected in 223 of the 237 countries, territories, and areas recognized by the World Health Organization [2]. Different regions have seen varying degrees of success with their specific mitigation strategies. Notably successful countries include Vietnam [3–5], New Zealand [6, 7], and Taiwan [8–10], which serve as important case studies for future pandemic preparedness efforts.

Other countries, including Canada, initially succeeded in controlling the spread of the virus, but went on to suffer a large second wave of infection amid reopening efforts [11, 12]. Even within Canada, COVID-19 mitigation success has varied by region. The Atlantic provinces of New Brunswick, Nova Scotia, and Prince Edward Island have been successful in controlling SARS-CoV-2 spread, with only small, occasional outbreaks that were rapidly contained [11, 13]. Other, larger, provinces, especially Ontario and Quebec, have struggled with critical periods of large and/or rapidly increasing known infection (KI) counts, responding with strict measures such as stay-at-home orders [14–16] and curfews [17].

**Fig. 1:**
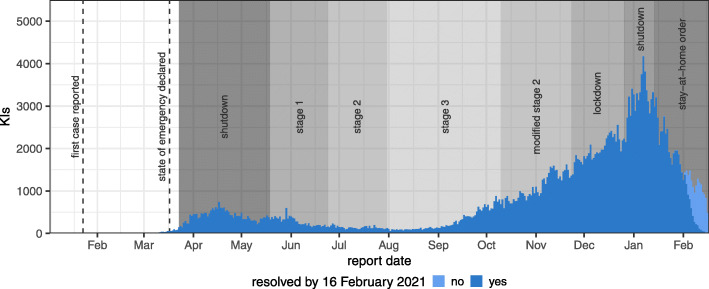
Known infections (KIs) over time in Ontario. Counts are split by whether or not the KI was resolved (marked as “resolved” or “fatal” in the Case and Contact Management database) by 16 February 2021 (see “Methods” section). Dashed vertical lines mark important dates for the outbreak in the province. Shaded regions indicate roughly when the most populous regions were in each reopening stage (reopening efforts have not been uniform across public health units). Larger stage numbers correspond to looser public health restrictions. Detailed descriptions of each reopening stage, recent shutdowns, and the newer zoned reopening framework can be found on the official Ontario COVID-19 website [16, 20, 25–28]

Our study is based on SARS-CoV-2 KI records in Ontario, where the first reported infection of SARS-CoV-2 was confirmed on 23 January 2020. The virus was detected sporadically in the province through February 2020 [18], until the number of KIs began to rise consistently in March 2020. Ontario declared its first pandemic-related state of emergency on 17 March 2020 [19], implementing a large-scale economic shutdown and school closures to mitigate spread of the virus. The province began reopening in stages through the summer [20] amid relatively low SARS-CoV-2 infection prevalence, with most schools reopening early-to-mid September [21]. In late September, additional restrictions were enacted across the province in response to the beginning of a second wave of infection [22–24], and on 23 November 2020 a lockdown was enacted in the heavily populated Toronto and Peel regions [25]. A province-wide shutdown began on 26 December 2020 [26], with an even stricter provincial stay-at-home order coming into effect on 14 January 2021, after KIs doubled in the preceding two weeks [27, 28]. The regions of Toronto, Peel, and North Bay-Parry Sound remain under this stay-at-home order as of 1 March 2021 [15], while the rest of the province follows a new zoned reopening framework [16]. Figure 1 summarises the time course of the SARS-CoV-2 epidemic in Ontario, as represented by KIs up to 16 February 2021.

One of the main motivations behind ongoing SARS-CoV-2 infection control efforts, in Ontario and around the world, is to ensure that hospitals do not become overwhelmed with a large demand for COVID-19 treatment. As well as compromising care of COVID-19 patients, a large demand for COVID-19 treatment would reduce access to and quality of care for many other conditions. With COVID-19 vaccination campaigns beginning around the world, policy-makers must seek to optimize vaccine distribution given limited supply. To protect individuals as well as the healthcare system, it is important to identify groups that are most likely to require significant medical care. Severity of COVID-19 presentation is highly variable among individuals, but increases with age [29–32]. Deaths attributed to COVID-19 have been found to be strongly concentrated in the elderly [33–35]. Comparatively few studies have explored the age distribution of serious medical interventions administered to COVID-19 patients [36, 37].

## Methods

The goal of this study is to quantify the relationships between COVID-19 patient age and the administration of serious medical interventions (hospitalizations, intensive care unit (ICU) admissions, intubations, and ventilations) for the province of Ontario. We compare these age-intervention associations with the age distributions of KIs and deaths. We also estimate the age-specific probability of hospitalization given known SARS-CoV-2 infection, and of survival given hospitalization related to COVID-19, to provide measures of individual risk.

### Data

We use individual-level records (line lists) for SARS-CoV-2 KIs reported from 23 January 2020 (the date of the first infection confirmation in Ontario) up to 16 February 2021 from the Case and Contact Management (CCM) database maintained by Public Health Ontario. This confidential central database includes detailed records of SARS-CoV-2 infections across the entire province of Ontario. These records include an individual’s demographic information (such as age), whether COVID-19-related interventions (including hospitalization, ICU admission, intubation, ventilation) were administered, as well as whether the infection was fatal.

The CCM database includes 288,532 KI records up to 16 February 2021. However, we analyse only KIs marked as “resolved” or “fatal” (*N*=277,555) to avoid tallying patients whose final outcomes are not yet known. KIs are marked as “resolved” in CCM based on public health unit assessment. In all instances, a record is considered resolved if it is 14 days past the symptom onset date (or specimen collection if symptom onset date is not known), though public health occasionally performs additional follow-up to update records. For brevity, we use “resolved KIs” in our analysis to refer to KIs marked as either “resolved” or “fatal” in CCM.

For population counts, we use 2020 Ontario population projections produced by Statistics Canada [38], specifically projections from the “M1” medium-growth scenario [39]. (All scenarios yield virtually identical projections for the short time horizon considered in this paper.)

We use provincial SARS-CoV-2 testing data from the Ontario Laboratories Information System (OLIS) database. This database records all tests for active SARS-CoV-2 infection performed through the provincial health system. These data are based on reports up to 16 February 2021 and include 270,402 positive tests and 9,163,489 negative tests.

We aggregate counts of all age-specific data (KIs, population, and tests) into two-year age bins, with one wide bin for individuals over 100 years old.

### Statistical models for probability estimates

To estimate the age-specific probabilities of hospitalization given KI, and of survival given hospitalization, we use two different approaches. In each case, we estimate a conditional probability of outcome *X* given that outcome *Y* has occurred and quantify uncertainty around this estimate using 95% confidence intervals.

The first approach is to consider each age group independently, and to assume that the number of times event *X* has occurred (given *Y*) follows a binomial distribution. We use the maximum likelihood estimate of the probability of outcome *X* given *Y*, which is simply the proportion of instances of *Y* where *X* has also occurred (points in Fig. 4). We then quantify the uncertainty around this point estimate by constructing a 95% Clopper-Pearson (exact) confidence interval (vertical bars in Fig. 4).

The second approach involves making the stronger assumption that there is a smooth relationship between age and the focal probability (curves in Fig. 4). We use a binomial generalized linear model (GLM) to estimate the age-specific survival probability given hospitalization [40]. This model assumes that probabilities follow a logistic curve as a function of age, and appears to be sufficient to quantify the monotonic relationship between survival probability and age. For the probability of hospitalization given KI, we use a binomial generalized additive model (GAM) [41], a generalization of the GLM that allows for non-monotonic trends. In particular, we fit a piecewise cubic spline (a penalized regression spline) to the log-odds as a function of age. Both of these parametric models give more precise results, and narrower confidence intervals (shaded bands in Fig. 4), than we obtain by computing probabilities and exact confidence intervals (age-by-age).

## Results

**Fig. 2:**
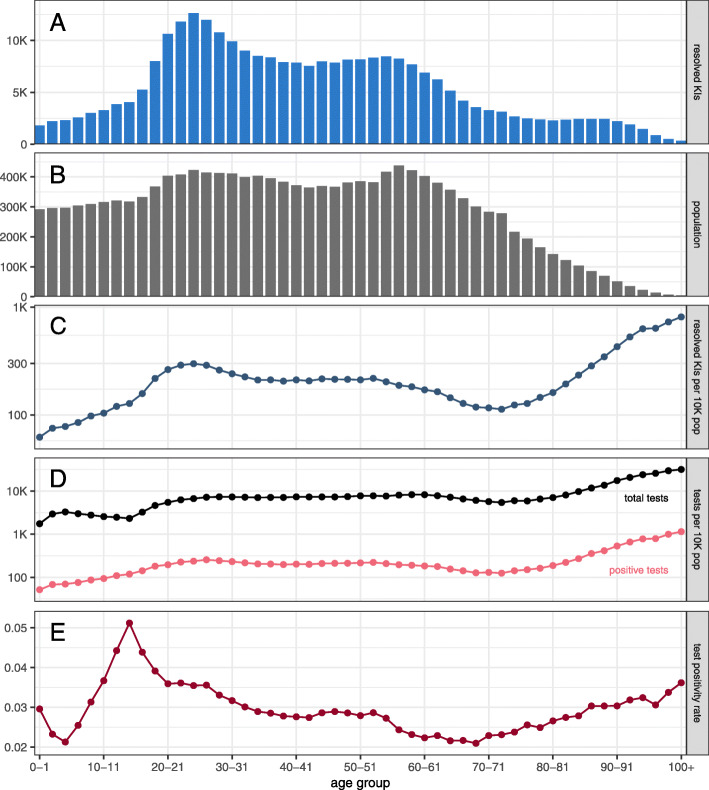
Age distribution of known infections (KIs) in Ontario. The distribution of ages for resolved KIs (panel **a**), Ontario population projections for 2020 (panel **b**), resolved KIs per 10,000 population (panel **c**), positive and total SARS-CoV-2 infection tests per 10,000 population (panel **d**), and test positivity rate (panel **e**). The test positivity rate is the proportion of tests administered that were positive. The *y*-axes in panels **c** and **d** are on a logarithmic scale

Figure 2 shows the age structure of resolved KIs, population demographics, and SARS-CoV-2 infection tests. The pattern observed in the raw counts of resolved KIs (panel a) reflects underlying demographics (panel b) as well as the testing intensity (panel d). Testing intensity, defined as the number of tests administered per 10,000 population by age group, increases sharply after age 75. The test positivity rate (panel e), defined as the proportion of tests administered that were positive, also reveals heterogeneities in testing across age groups, peaking in 14–15-year-olds. Controlling for demography, the number of detected infections (panel c) is comparatively low in ages under 15, increases to a plateau for ages 20–70 (with a noticeable peak around age 25), and then continually increases after age 70. The number of resolved KIs per capita is 1.43 times higher in ages 20–29 than in ages 30–69.

**Fig. 3:**
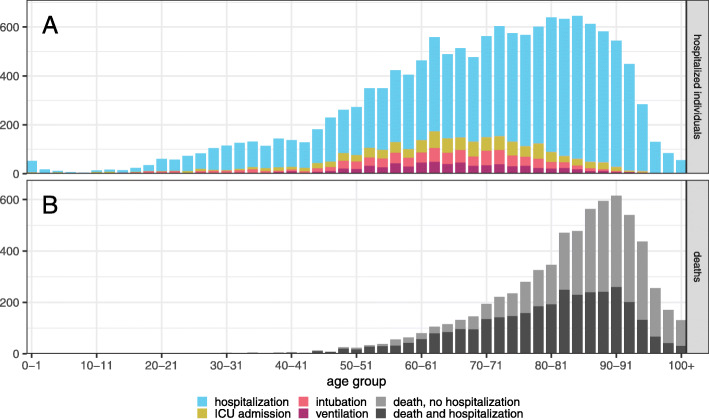
COVID-19 outcomes by age in Ontario. The distribution of ages for hospital interventions (panel **a**), and deaths (panel **b**). Hospital outcomes are nested and tallied by the most intensive medical intervention used for each patient (ventilator use is the most intensive, followed by intubation, ICU admission, and hospitalization). Deaths are split by whether or not the patient also had a record of hospitalization for COVID-19 treatment

Figure 3 shows the distribution of ages for serious medical interventions (panel a) and deaths (panel b) related to COVID-19 for resolved KIs. We present raw counts, as opposed to counts normalized by the age-specific population, because the raw counts (not per capita counts) determine the pressure on the healthcare system. Hospitalizations are split by the most intensive known intervention (with ventilator use being the most intensive, followed by intubation, then ICU admission, then hospitalization). Deaths are split by whether or not the patient has a record of hospitalization for COVID-19 treatment.

**Fig. 4:**
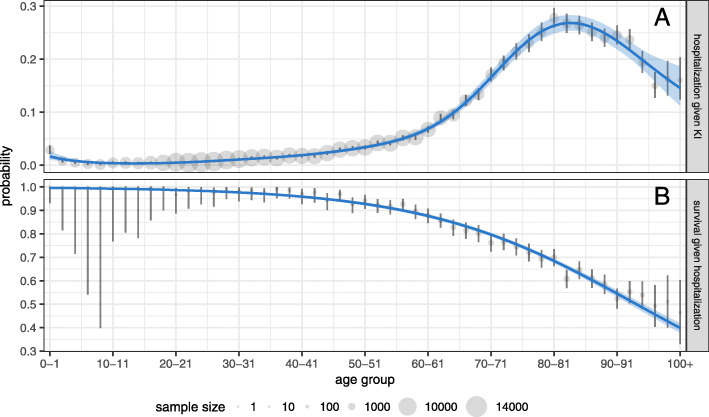
Age-dependent COVID-19 hospitalization probability for known SARS-CoV-2 infection (panel **a**) and survival probability for hospitalized patients (panel **b**) in Ontario. We give age-by-age estimates of each probability (points; 95% exact binomial confidence intervals given by vertical lines), where point area is proportional to age-specific sample size. We additionally provide more precise estimates of these probabilities under stricter assumptions, modelling the hospitalization probability using a generalized additive model and the survival probability using a generalized linear model (curves; 95% confidence bands given by shaded regions). See “Methods” section for details

The distribution of serious medical interventions is much wider than that of deaths, with the latter peaking at age 90. Hospitalizations are relatively uniformly spread between ages 60–90, while the distribution of ICU-related interventions (ICU admission, intubation, ventilation) is spread over a slightly younger age range. The total number of COVID-19 deaths in Ontario up to 16 February 2021 was 6,728, of which 3,475 (51.6%) had no record of hospitalization for treatment related to COVID-19.

Figure 4 shows the estimated hospitalization probability given known SARS-CoV-2 infection (panel a) and survival probability given hospitalization for COVID-19 treatment (panel b). These metrics quantify individual risk of serious COVID-19 outcomes as a function of age (without explicitly controlling for other factors like comorbidities; see “Limitations” section). The large uncertainty in age-by-age probability estimates for some young and very old age groups is due to small numbers of KIs and hospitalizations in these ages. However, the estimated uncertainty is much lower with the model-based approach (see Methods: “Statistical models for probability estimates” section). Based on age-by-age estimates, the hospitalization probability peaks in the 80–81 age group at 27.8% (95% CI 26.0%–29.7%). In adults, the survival probability is near 100% until about age 40, where it begins to decline steadily. For instance, a hospitalized individual in the age group 54–55 has a 91.7% chance of surviving COVID-19 (95% CI 88.3%–94.4%; according to age-by-age-estimates), implying that nearly 1 in 12 hospitalized COVID-19 patients in this age group die despite receiving acute care for the illness.

## Discussion

The age distributions of known SARS-CoV-2 infections (Fig. 2a) and deaths attributed to COVID-19 (Fig. 3b) on their own provide limited insight into the risk that COVID-19 patients could overwhelm Ontario’s healthcare system. The majority of COVID-19-related deaths have occurred in patients with no record of hospitalization (Fig. 3b). Many deaths have occurred in long-term care (LTC) facilities, which are independent of the hospital system [42], and have experienced significant outbreaks [42, 43] (Fig. 2c), necessitating considerable disease surveillance in very old age groups (Fig. 2d). (These LTC deaths may partially explain the observed decrease in the hospitalization probability after age 80, Fig. 4a.) Unlike the distribution of deaths (Fig. 3b), the broad age distributions of hospitalizations, ICU admissions, intubation, and ventilation (Fig. 3a) reveal the potential pressure on the healthcare system from both middle-aged individuals and seniors.

Early in the outbreak, the province of Ontario expanded coverage for COVID-19-related treatment to include even individuals who are not usually covered by the Ontario Health Insurance Plan [44]. Access to prompt and successful medical interventions may have kept a large proportion of COVID-19-related hospitalizations from resulting in deaths. While we expect that the shape of the age distribution of the *need* for hospitalization (Fig. 3a) would remain the same if prevalence were to increase significantly, the distribution of deaths (Fig. 3b) may expand toward younger ages if hospitals and ICUs reach maximum capacity (due to an insufficient supply of acute care).

This potential need for acute care, coupled with elevated individual risks associated with COVID-19 in the same age range (Fig. 4), support the prioritization of infection prevention in young seniors and middle-aged individuals (in addition to old seniors) when designing vaccine distribution strategies. However, this result does not necessarily imply that an age-based “oldest-to-youngest” vaccination strategy is optimal to achieve the goal of preventing infection in these groups. A recent study by Mulberry et al. [45] suggests that overall vaccination strategies prioritizing essential workers can indirectly protect the most vulnerable groups and outperform oldest-to-youngest vaccination strategies by reducing the number of KIs, hospitalizations, deaths, instances of “long COVID” [46], and by increasing net economic benefit. Vaccination strategies targeting groups most likely to transmit the disease (with the goal of protecting vulnerable groups) have been explored previously in the context of seasonal influenza [47]. For COVID-19, strategies prioritizing groups most likely to transmit the virus are especially effective when the vaccine has high efficacy (in terms of reducing susceptibility to infection) [48], which is true of several leading COVID-19 vaccines [49–52].

The age-dependent probabilities of hospitalization given KI (Fig. 4a) are based on resolved *known* infections, and so they depend on how widely SARS-CoV-2 testing has been conducted. Throughout the period covering a large portion of the CCM data, testing guidelines selected for sufficiently symptomatic individuals [53]. These guidelines were not expanded to include asymptomatic individuals from the general public until 29 May 2020 [54] and were rolled back on 24 September 2020 in an effort to preserve limited testing resources amid a surge in KIs [55]. As a result, untargeted asymptomatic testing was offered only in the summer, when prevalence was relatively low, which represents a small proportion of the data. Moreover, individuals may not be prompted to get tested in the absence of symptoms unless they are included in a contact tracing investigation. The probability of hospitalization given KI therefore likely overestimates the underlying probability of hospitalization given infection, whether known or not.

Our survival probability estimates for hospitalized individuals (Fig. 4b) are *not* affected by the same detection biases present in KI data. Patients admitted to hospital are tested for SARS-CoV-2 as part of infection control protocols, and thus infection detection in hospitalized individuals is not influenced by testing guidelines for the general population. Our survival probability estimates do, however, represent an upper bound with respect to the current standard of care and viral variant. In the absence of significant innovation in COVID-19 treatment or viral evolution to lower disease severity, we expect survival probabilities would decrease if ICUs or hospitals were to reach maximum capacity.

### Limitations

Age is a simple and accessible proxy for risk factors, including existing comorbidities that may affect COVID-19 outcomes, which on average scale with age. This study did not explicitly account for comorbidities and other factors that could correlate with the severity of COVID-19 outcomes.

In general, KIs underestimate the true prevalence of SARS-CoV-2 infection for a variety of reasons, including test availability, ease of testing, test accuracy, and difficulties in detecting asymptomatic individuals. The majority of KIs captured in the data analysed in this study occurred when testing guidelines were selecting for sufficiently symptomatic individuals, and so asymptomatic and mild infections are likely underrepresented.

This study is specific to the Ontario SARS-CoV-2 epidemic, though the results have implications for COVID-19 outbreaks all over the world. Contact patterns in Ontario have changed over the course of the pandemic due to the province’s continuing effort to control COVID-19 spread while also supporting the economy. Observed patterns in the age distributions of KIs and deaths may change as the age-specific contact structure and contact rates continue to change.

## Conclusions

We have quantified the age distributions of serious medical interventions for SARS-CoV-2 infection in Ontario, Canada, for the entire period of the regional epidemic through 16 February 2021. Our results reveal a large need for hospitalization in a broad age range (mainly ages 60-90): a threat of the ongoing COVID-19 pandemic that is not revealed by the age distribution of KIs and deaths alone. If healthcare capacities were to be exceeded due to an increase in prevalence, the need for COVID-19-related acute care may not be met adequately, which could expand the existing distribution of deaths toward younger ages. Moreover, the probability of survival given COVID-19-related hospitalization is lower than is generally perceived for patients over 40. Vaccination programs prioritizing older age groups to prevent deaths should consider broadening their priorities to also prevent infection in younger seniors and middle-aged individuals, in order to help ensure the healthcare system does not exceed its capacity for acute care.

The Government of Canada and the Province of Ontario have implemented policies meant to help mitigate SARS-CoV-2 spread while also undertaking a phased reopening [16, 20, 56]. Future work should consider whether the age dependence of SARS-CoV-2 infection risks is changing over time, as the population continues to navigate the pandemic and the testing effort expands. Our study explores only short-term SARS-CoV-2 infection outcomes; future studies should explore the age distributions of long-term morbidities from this infection, so that we may better understand the heterogeneous risks associated with COVID-19. Lastly, all studies relying on KI counts are subject to bias from how infections are detected via active infection testing. Future work should seek to correct for this bias.

## Data Availability

Aggregate data and source code required to reproduce all analyses presented in this study are available at https://github.com/papsti/covid-age. Declarations

## Abbreviations

SARS-CoV-2: Severe Acute Respiratory Syndrome Coronavirus 2
COVID-19: Coronavirus Disease 2019
KI: Known infection, ICU: Intensive Care Unit
CCM: Case and Contact Management
OLIS: Ontario Laboratories Information System
GLM: Generalized linear model
GAM: Generalized additive model
LTC: Long-Term Care
